# Supplementary material for: Aqueous pulsed electrochemistry promotes C−N bond formation via a one-pot cascade approach
Source: Nat Commun. 2023 Aug 22;14:5088. doi: 10.1038/s41467-023-40892-9 (PMC10444869; doi:10.1038/s41467-023-40892-9)
Supplement: Supplementary file 1 — Supplementary Information [file 41467_2023_40892_MOESM1_ESM.pdf]

Contents

Supplementary Figure 1. The pathway for the formation of phenol from arylboronic acids.
Supplementary Figure 2. SEM images and XRD pattern of $Cu(OH)_2$ NWAs.
Supplementary Figure 3. CV curve of the Cu_2O NCs.
Supplementary Figure 4. Experimental and simulated XAFS spectra of LC-Cu at the Cu *K*-edge.
Supplementary Figure 5. ECSA tests of different Cu nanocatalysts.
Supplementary Figure 6. Comparison of the performance via NO_2^- reduction over LC-Cu NCs and Cu foam.
Supplementary Figure 7. *j-t* profile of LC-Cu NCs induced by the pulsed-potential protocol.
Supplementary Figure 8. The concentration-absorbance calibration curves of ammonia-N.
Supplementary Figure 9. Time-dependent amount of substance change of NO_2^- and NH_3 at −1.1 V.
Supplementary Figure 10. LSV curves of LC-Cu NCs with aniline **2a** and ammonia.
Supplementary Figure 11. Yield of NH_3 under pulsed electrolysis conditions.
Supplementary Figure 12. The result of GC–MS after subjecting **2a** to pulsed electrolysis.
Supplementary Figure 13. ^{1}H NMR spectra of the electrolyte.
Supplementary Figure 14. In situ ATR-FTIR tests of NO_2^-RR.
Supplementary Figure 15. UV spectra of Cu(II) under different reaction conditions.
Supplementary Figure 16. Yields of phenol byproduct under different pH conditions without electricity.
Supplementary Figure 17. UV spectra of Cu(II) and images of the electrode under different conditions.
Supplementary Figure 18. Characterizations of LC-Cu NCs after pulsed electrolysis.
Supplementary Figure 19. ^{15}N abundance of Aniline-^{15}N.
Supplementary Figures 20~35. ^{1}H and ^{13}C NMR spectra of products.
Supplementary Note 1. C–O bond formation of Chan-Lam reaction.
Supplementary Note 2. Analysis of the SEM and XRD results of $Cu(OH)_2$ NWAs.
Supplementary Note 3. Analysis of CV curves results of Cu_2O NCs.
Supplementary Note 4. Reactions for the electroreduction of NO_2^- to NH_3 over LC-Cu NCs.
Supplementary Note 5. Reaction condition of pulsed step.
Supplementary Note 6. Analysis of time-dependent transformations of NO_2^- to NH_3.
Supplementary Note 7. Analysis for the LSV curves of ammonia and **2a** over LC-Cu NCs.

Supplementary Note 8. Experiment detailes for the electroreduction of NO_2^- to NH_3.

Supplementary Note 9. Analysis of the results of GC–MS.

Supplementary Note 10. NMR spectra of $^{14}NH_4^+$ and $^{15}NH_4^+$.

Supplementary Note 11. Analysis of the results of in situ ATR-FTIR tests of NO_2^-RR.

Supplementary Note 12. Analysis of the results of UV spectra.

Supplementary Note 13. Analysis for the yield of byproducts under different pH value.

Supplementary Note 14. Analysis of the concentration of Cu(II) after pulsed electrolysis and potentiostatic electrolysis.

Supplementary Note 15. The results for the characterizations of LC-Cu NCs after pulsed electrolysis.

Supplementary Note 16~31. NMR spectra analyses of the products.

Supplementary Table 1. EXAFS fitting parameters at the Cu *K*-edge.

Supplementary Table 2. Reaction Development.

Supplementary Table 3. Substrate scope for the electrocatalytic transfer of arylboronic acids and aryl Bpin to primary arylamine in our system.

Supplementary References (1-3)

Supplementary Figures and Notes

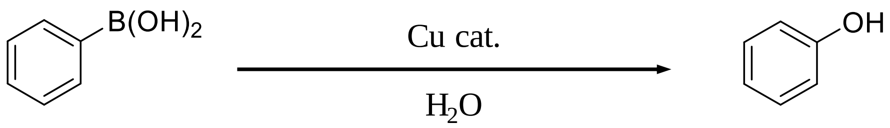

Supplementary Fig. 1 The pathway for the formation of phenol from arylboronic acids.

Supplementary Note 1 A previous report demonstrated that the presence of water in the system causes oxidation, a competitive Chan–Lam C–O bond formation that uses water as the heteroatomic nucleophile.[1]

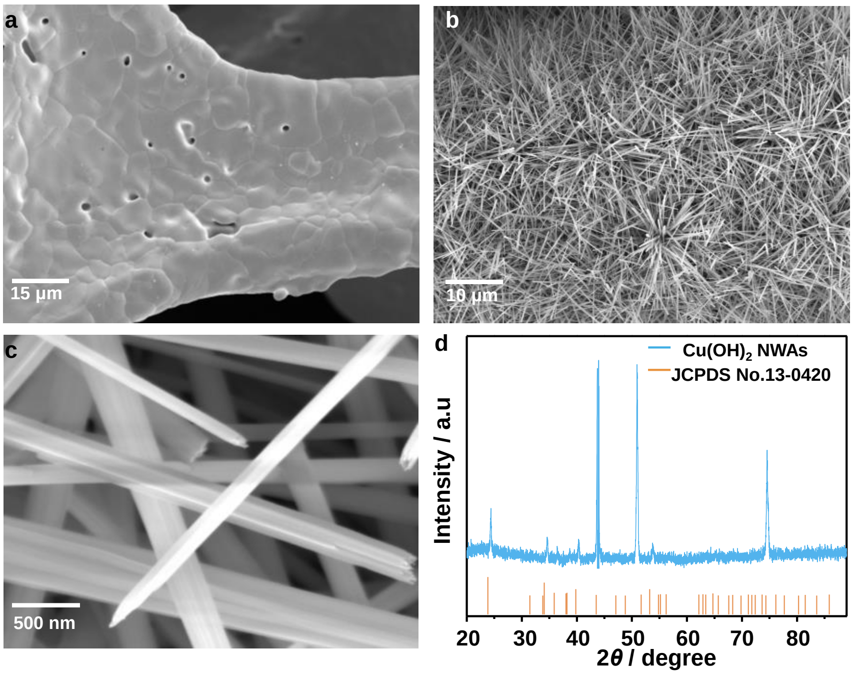

Supplementary Fig. 2 SEM images and XRD pattern of $Cu(OH)_2$ NWAs. a A low magnification SEM image of bare Cu foam. Low magnification **b** and high magnification SEM images **c** of $Cu(OH)_2$ NWAs. **d** X-ray diffraction (XRD) patterns of $Cu(OH)_2$ NWAs.

Supplementary Note 2 SEM images reveal that $Cu(OH)_2$ NWAs grew uniformly on the Cu foam. All the diffraction peaks in the XRD pattern could be indexed to $Cu(OH)_2$ (JCPDS NO.13-0420).[2]

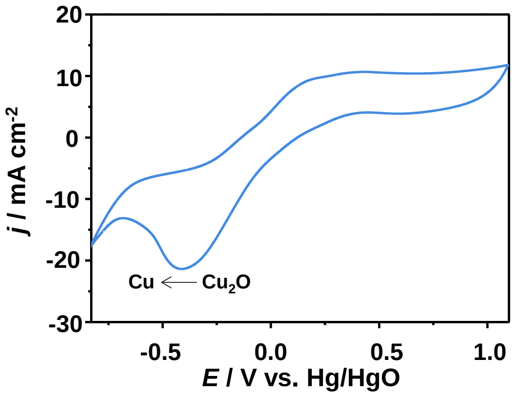

Supplementary Fig. 3 CV curve of the Cu_2O NCs obtained at a scan rate of 1 mV s^{-1} in a mixed solution of 0.25 M PBS and MeOH (2:1 *v*/*v*).

Supplementary Note 3 The CV curve displays a reduction peak at −0.45 V vs. Hg/HgO, corresponding to Cu(I)/Cu(0).

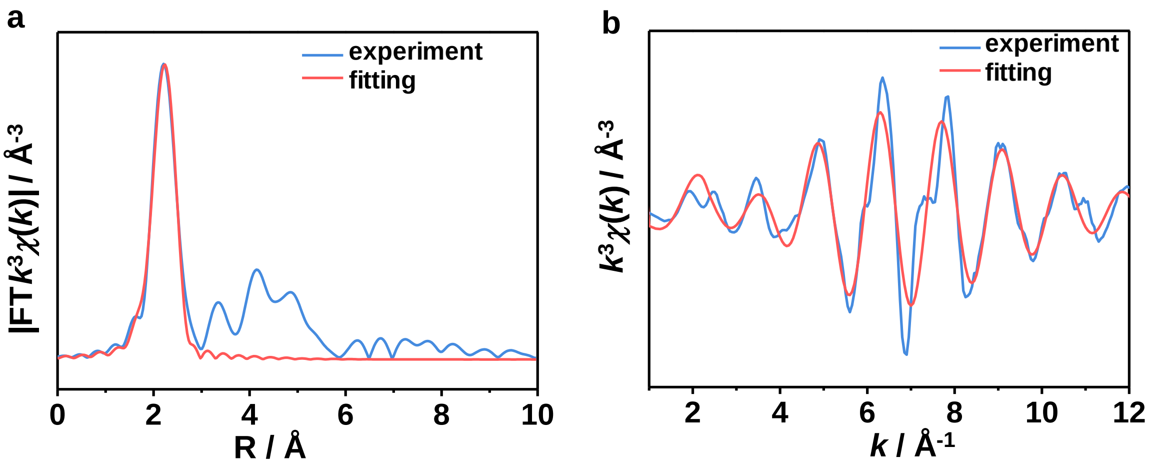

Supplementary Fig. 4 Experimental and simulated XAFS spectra of LC-Cu at the Cu *K*-edge. a Fourier transform (FT) to R-space to isolate the EXAFS contributions from each coordination shell. **b** The χ(k) data weighted by k^3. Quantified fitting results are shown in Supplementary Table 1.

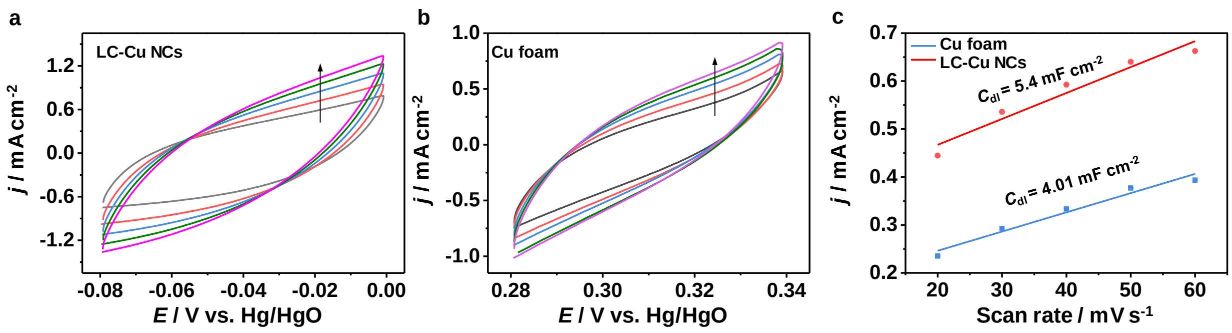

Supplementary Fig. 5 ECSA tests of different Cu nanocatalysts. CV curves of **a** LC-Cu NC and **b** Cu foam with various scan rates from 20 to 60 mV s^{-1}. **c** The double layer capacitance (C_{dl}) of different catalysts.

$$A_{ECSA}^{Cu\ NCs} = \frac{5.4\ mF\ cm^{-2}}{40\mu F\ cm^{-2}\ per^{2}cm^{2}_{ECSA}} = 135\ cm^{2}_{ECSA}$$

$$A_{ECSA}^{Cu\ foam} = \frac{4.01\ mF\ cm^{-2}}{40\mu F\ cm^{-2}\ per^{2}cm^{2}_{ECSA}} = 100.25\ cm^{2}_{ECSA}$$

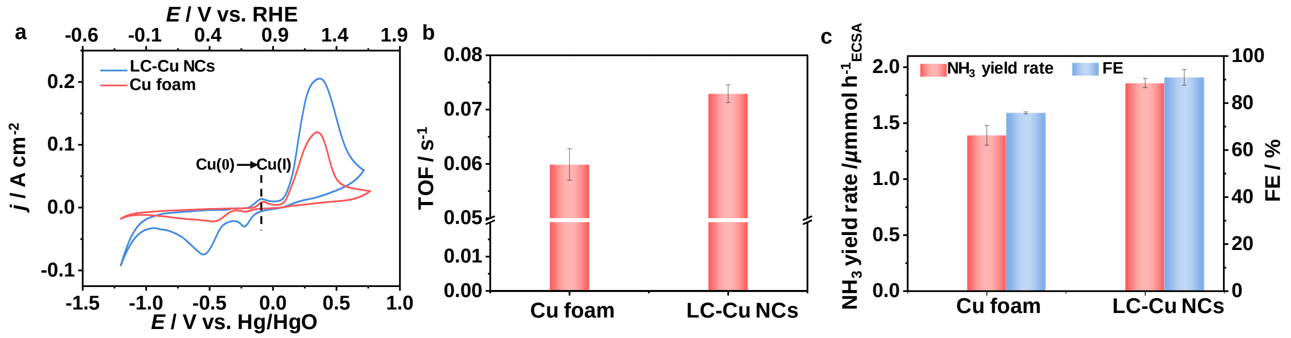

Supplementary Fig. 6 Comparison of the performance via NO_2^- reduction over LC-Cu NCs and Cu foam. **a** CV curves of LC-Cu NCs and Cu foam at a scan rate of 5 mV s^{-1}. **b** TOF values and **c** yield rate and FE of NH_3 via NO_2^- reduction over LC-Cu NCs and Cu foam in a mixed solution of 0.25 M PBS and MeOH (2:1 *v*/*v*) at −1.1 V.

Supplementary Note 4 As shown in Supplementary Fig. 6b and Fig. 6c, the performance of the electroreduction of NO_2^- to NH_3 over LC-Cu NCs is much better than that over Cu foam.

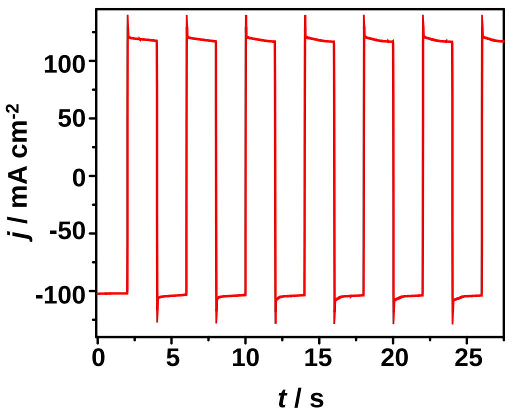

Supplementary Fig. 7 *j-t* profile of LC-Cu NCs induced by the pulsed-potential protocol.

Supplementary Note 5 Transient current signals recorded as a result of the discrete, step-like alternation between E_{an} and E_{ca} during the pulsed potential protocol: $E_{ca} = -1.1$ V, $E_{an} = 0.4$ V, $t_{ca} = t_{an} = 2$ s.

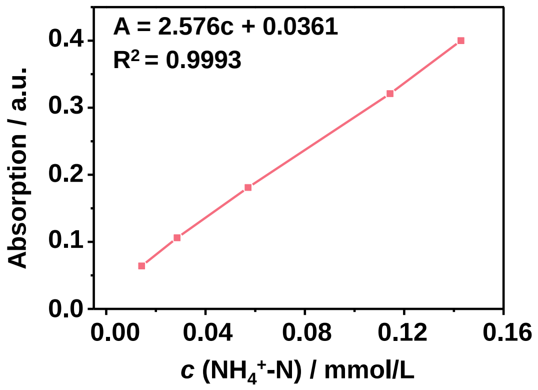

Supplementary Fig. 8 The concentration-absorbance calibration curves of ammonia-N.

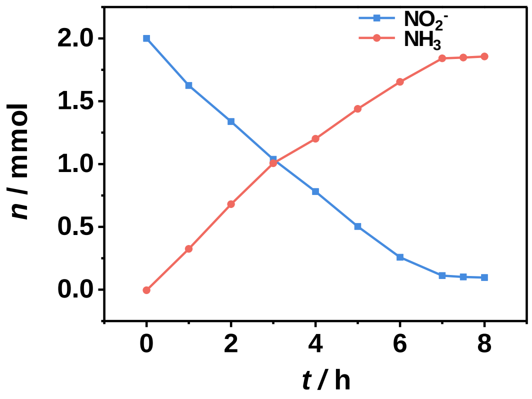

Supplementary Fig. 9 Time-dependent amount of substance change of NO_2^- and NH_3 over LC-Cu NCs at −1.1 V.

Supplementary Note 6 Time-dependent transformations indicate that nearly full conversion of NO_2^- is finished in 7 h.

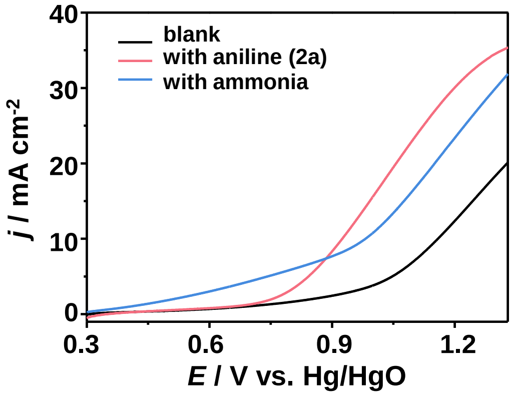

Supplementary Fig. 10 LSV curves of LC-Cu NCs at a scan rate of 5 mV s^{-1} in a mixed solution of 0.25 M PBS (pH = 12) and MeOH (2:1 *v*/*v*) with aniline 2a and ammonia.

Supplementary Note 7 The LSV curves of LC-Cu NCs demonstrated that ammonia and **2a** were more easily oxidized than the OER, and a much lower potential was required for ammonia oxidation than that of **2a**.[3]

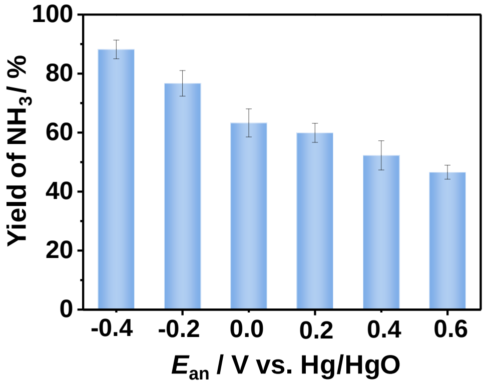

Supplementary Fig. 11 Yield of NH_3 under pulsed electrolysis conditions with E_{ca} = −1.1 V, different E_{an} values, and t_{an} = t_{ca} = 2 s.

Supplementary Note 8 After the electrochemical experiments were carried out under potentiostatic conditions for 7 hours to reduce nitrite to ammonia, the yield of NH_3 decreased at the higher anodic potential (E_{an}).

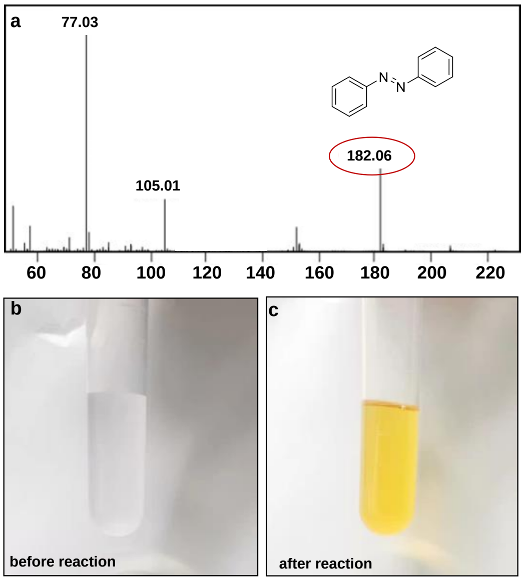

Supplementary Fig. 12 The result of GC–MS after subjecting 2a to pulsed electrolysis. a GC–MS test, and **b** and **c** the color change of the reaction mixture when subjecting **2a** to pulsed electrolysis ($E_{ca} = -1.1$ V, $E_{an} = 0.6$ V, $t_{ca} = t_{an} = 2\ s$) for 5 h.

Supplementary Note 9 We observe a rapid color change in the solution and detect the azobenzene product by GC–MS when subjecting **2a** to pulsed electrolysis with an E_{an} of 0.6 V, indicating the oxidation of **2a**.

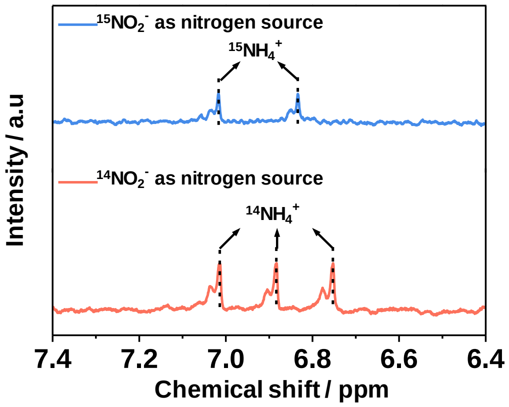

Supplementary Fig. 13 ^{1}H NMR spectra of the electrolyte after the electrocatalytic NO_2^- reaction using $^{14}NO_2^-$ and $^{15}NO_2^-$ nitrogen sources.

Supplementary Note 10 The ^{1}H NMR spectra of the electrolyte after the electrocatalytic reduction of $Na^{15}NO_2$ show the typical double peaks of $^{15}NH_4^+$ at $\delta = 6.84$ and 7.02 ppm, demonstrating that the synthesis of NH_3 resulted from the electroreduction of NO_2^-.

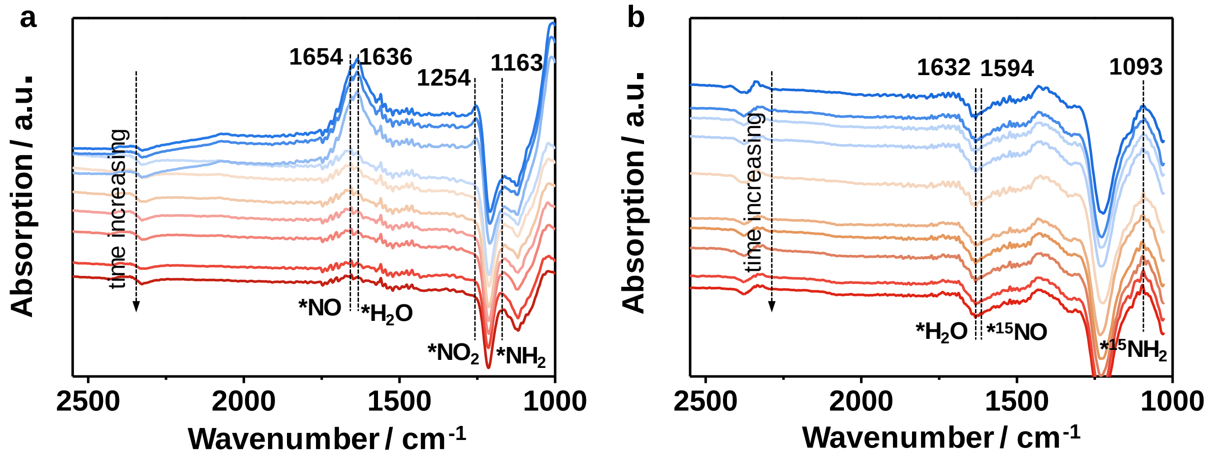

Supplementary Fig. 14 In situ ATR-FTIR tests of NO_2^-RR. Isotope-labelling electrochemical in situ ATR-FTIR spectra of LC-Cu NCs using **a** $^{14}NO_2^-$ and **b** $^{15}NO_2^-$.

Supplementary Note 11 As the results show, the peak located at 1254 cm^{-1} is assigned to $*NO_2$ and declines progressively owing to the conversion of NO_2^- as electrolysis proceeds. The peaks located at 1654 cm^{-1} and 1163 cm^{-1} belong to *NO and $*NH_2$, which are the key intermediates during NO_2^- reduction. Additionally, these shifted to lower wavenumbers for $*^{15}NO$ (1594 cm^{-1}) and $*^{15}NH_2$ (1093 cm^{-1}) due to the isotope effect.

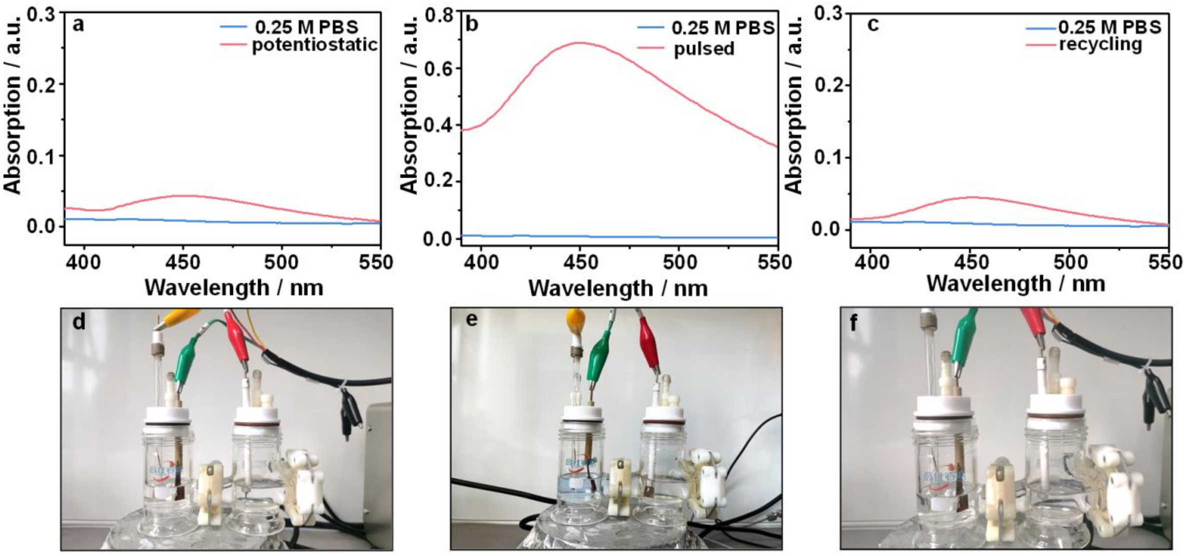

Supplementary Fig. 15 UV spectra of Cu(II) under different reaction conditions. UV spectra of Cu(II) under different reaction conditions and corresponding images of the reaction setup: **a** and **d** after potentiostatic electrolysis, **b** and **e** after pulsed electrolysis, and **c** and **f** after pulsed electrolysis and then under potentiostatic electrolysis for 20 mins.

Supplementary Note 12 Supplementary Fig. 15 displays that nearly no Cu(II) is detected under the potentiostatic electrolysis reaction. After pulsed electrolysis ($E_{ca} = -1.1$ V, $E_{an} = 0.4$ V, $t_{ca} = t_{an} = 2$ s) for 12 h, we observe the presence of Cu(II) from the UV–Vis absorption peak due to the leaching of Cu(II) from the Cu electrode under the pulsed anodic potential, and a clear blue color is also seen and combined with EPR, which may be due to the dissolution of the formed Cu(II)-NH_3 complex in the solution during the reaction. Then, after the pulsed electrolysis is finished, we conduct potentiostatic electrolysis at −1.1 V for 20 mins. The UV–Vis absorption peak is significantly weakened, and the blue color vanishes, indicating that there is almost no Cu(II) in the solution. This shows that Cu(II) can be well recovered via electrochemical deposition, thus reducing the detriment of Cu residuals to the products and environment.

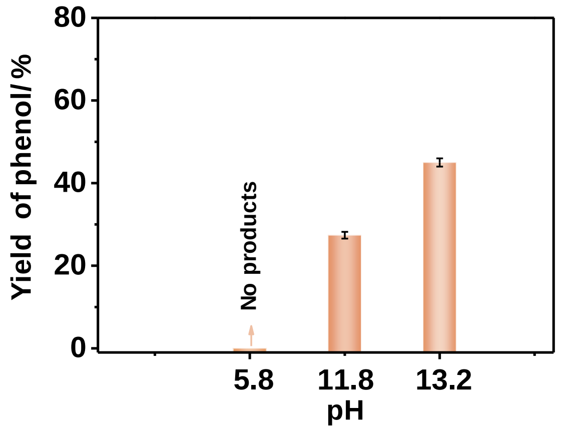

Supplementary Fig. 16 Yields of phenol byproduct under different pH conditions without electricity (0.01 mmol of phenylboronic acid (**1a**), 0.03 mmol of $Cu(OAc)_2 \cdot H_2O$ as the catalyst, 0.25 M PBS (pH = 5.8, 11.8 and 13.2) and MeOH (2:1 *v*/*v*).

Supplementary Note 13 The compared results show that a higher pH value is favorable for the formation of phenol byproducts.

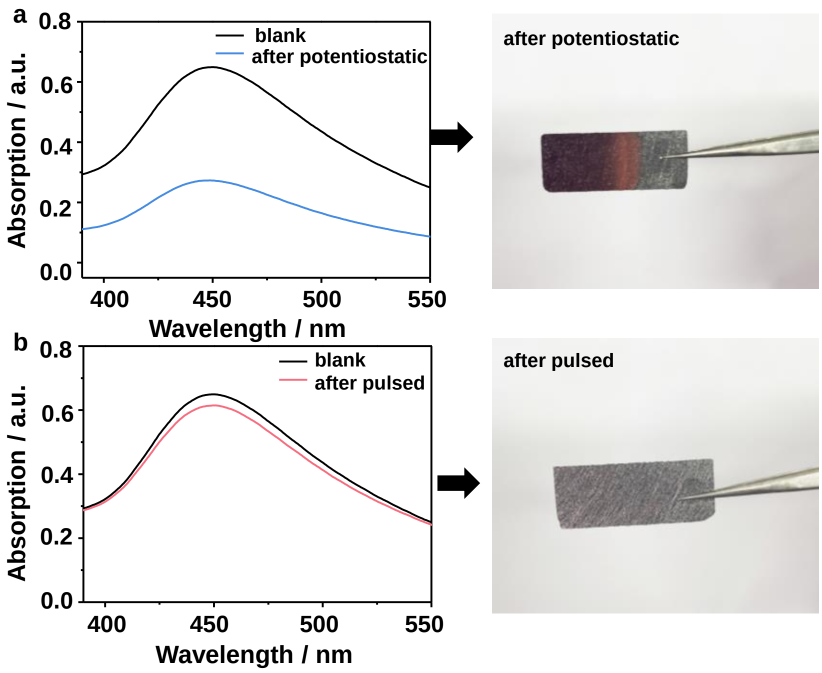

Supplementary Fig. 17 UV spectra of Cu(II) and images of the electrode under different conditions. UV spectra of Cu(II) and images of the electrode after subjecting 0.01 mmol of Cu(OAc)$_2$ H$_2$O to **a** potentiostatic electrolysis at −1.1 V, and **b** pulsed electrolysis (E_{ca} = −1.1 V, E_{an} = 0.4 V, t_{ca} = t_{an} = 2 s) for 20 mins by using carbon paper (CP) as the working electrode, 0.25 M PBS (pH = 12) and MeOH (2:1 *v*/*v*) as the electrolyte.

Supplementary Note 14 The concentration of Cu(II) decreases much less after pulsed electrolysis than that under potentiostatic electrolysis, and nearly no Cu is deposited on the surface of CP. These results demonstrate that Cu(II) can remain stable for a longer period under pulsed conditions, offering a great opportunity for long-term electrolysis.

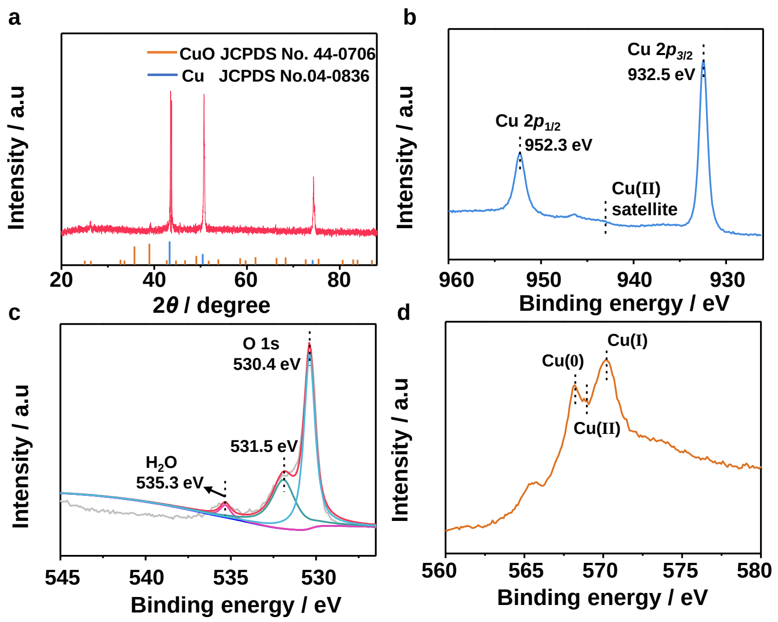

Supplementary Fig. 18 Characterizations of LC-Cu NCs after pulsed electrolysis. a XRD patterns, **b** and **c** XPS spectra, and **d** Cu LMM AES spectra of LC-Cu NCs after pulsed electrolysis.

Supplementary Note 15 The above results reveal that copper oxides are formed on the Cu surface after pulsed electrolysis.

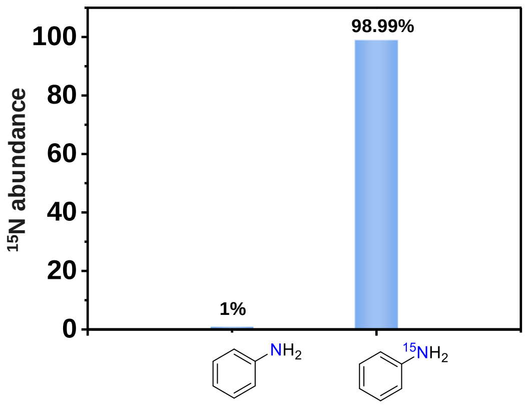

Supplementary Fig. 19 ^{15}N abundance of Aniline-^{15}N.

Supplementary Table 1 EXAFS fitting parameters at the Cu *K*-edge for various samples (S_0^2=0.837)

Sample	Path	C.N.	R (Å)	σ^2 (Å^2)	ΔE (eV)	R factor
Cu foam	Cu-Cu	12*	2.54±0.01	0.0085	4.34±0.6	0.0052
LC-Cu	Cu-Cu	9.75±0.8	2.54±0.01	0.0094	3.64±0.9	0.0131

[a]*C.N.* : coordination numbers; [b]*R*: bond distance; [c]σ^2: Debye-Waller factors; [d] Δ*E*: the inner potential correction.

Supplementary Table 2 Reaction Development[a,b]

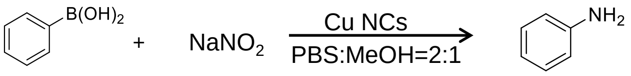

entry	condition	yield %
1	$NaNO_2$ (1.0 mmol)	25
2	$NaNO_2$ (2.0 mmol)	72
3	$NaNO_2$ (5.0 mmol)	53
4	$NaNO_2$ (2.0 mmol), Ar	70
5[b]	$NaNO_2$ (2.0 mmol), $Cu(OAc)_2$ (0 mmol)	trace
6[b]	$NaNO_2$ (2.0 mmol), $Cu(OAc)_2$ (0.01 mmol)	35
7[b]	$NaNO_2$ (2.0 mmol), $Cu(OAc)_2$ (0.03 mmol)	42
8[b]	$NaNO_2$ (2.0 mmol), $Cu(OAc)_2$ (0.06 mmol)	27

Reaction conditions: Phenylboronic acid (**1a**, 0.1 mmol), $NaNO_2$, Hg/HgO as the reference electrode, pulsed potential conditions (E_{ca} = −1.1 V, E_{an} = 0.4 V, t_{ca} = t_{an} = 2 s), 0.25 M PBS (pH = 5.8) and MeOH (2:1 *v*/*v*), an H-type electrolytic cell, air, rt. [b]Constant potential (−1.1 V, 12 h) with different amounts of $Cu(OAc)_2$ H_2O, 12 h.

Supplementary Table 3 Substrate scope for the electrocatalytic transfer of arylboronic acids and aryl Bpin to primary arylamine in our system.

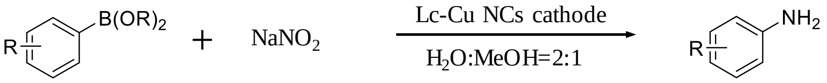

1 **2**

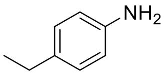	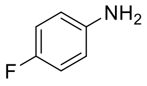	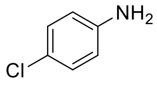	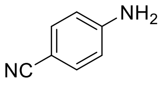	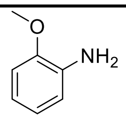
**2d**, 83%	**2e**, 51%	**2f**, 60%	**2g**, 53%	**2h**, 70%
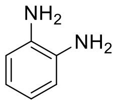	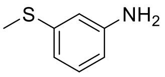	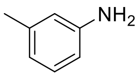	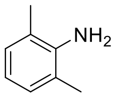	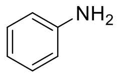
**2i**, 74%	**2j**, 50%	**2k**, 63%	**2l**, 52%	**2o**, 62%[b]

[a]Conditions: Ar-$B(OH)_2$ (0.1 mmol), $NaNO_2$ (2 mmol), 0.25 M PBS and MeOH (2:1 *v*/*v*, 24 mL), LC-Cu NCs (working area: 1 cm^2), air, rt. Isolated yields are reported. [b] Using Ar-BPin as the substrate.

Supplementary NMR spectra and GC–MS data

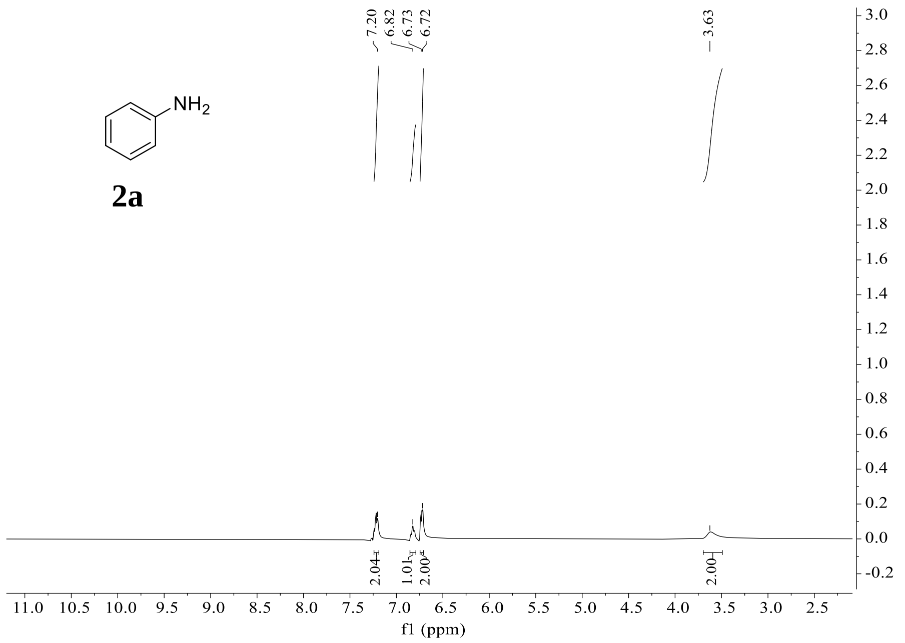

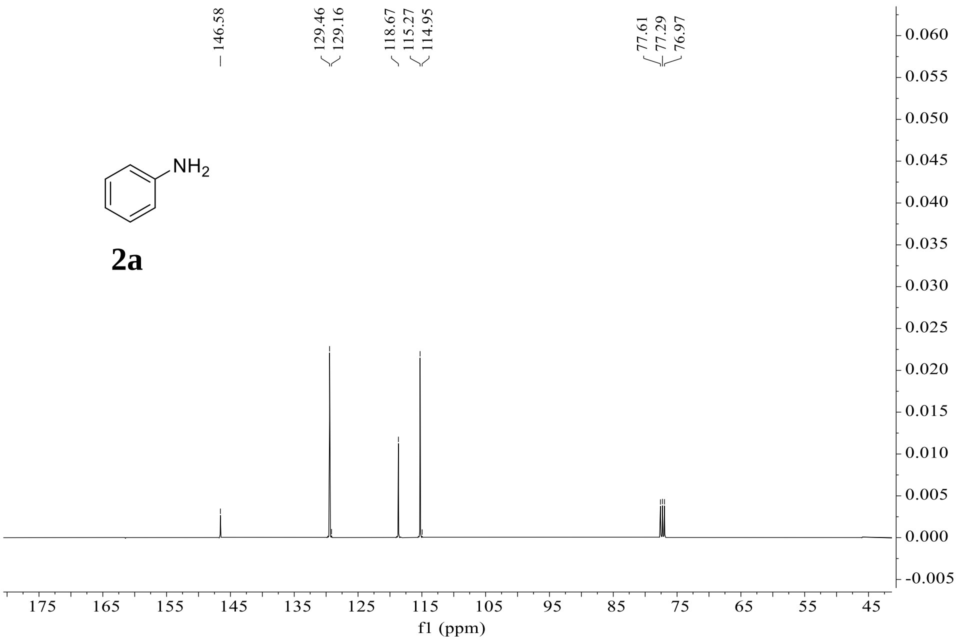

Supplementary Fig. 20 ^{1}H NMR and ^{13}C NMR spectrum of aniline.

Supplementary Note 16 ^{1}H NMR (400 MHz, $CDCl_3$) δ [ppm] 7.20 (s, 2H), 6.82 (s, 1H), 6.73 (d, J = 4.0 Hz, 2H), 3.63 (s, 2H); ^{13}C NMR (101 MHz, $CDCl_3$) δ [ppm] 146.58, 129.46, 129.16, 118.67, 115.27, 114.95; GC–MS (EI) m/z 93.1, theoretical value for $C_6H_5NH_2$ is 93.12.

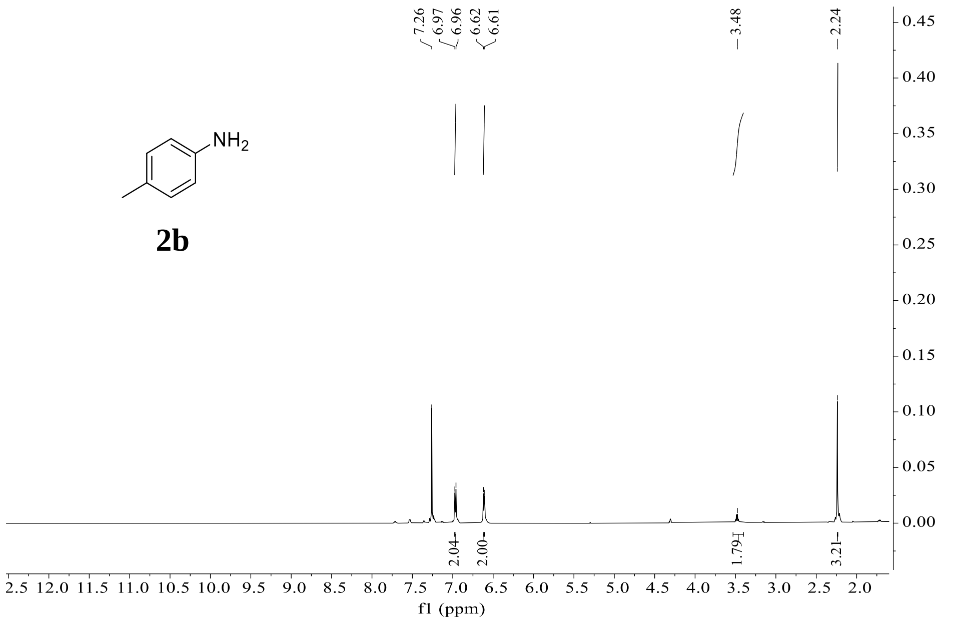

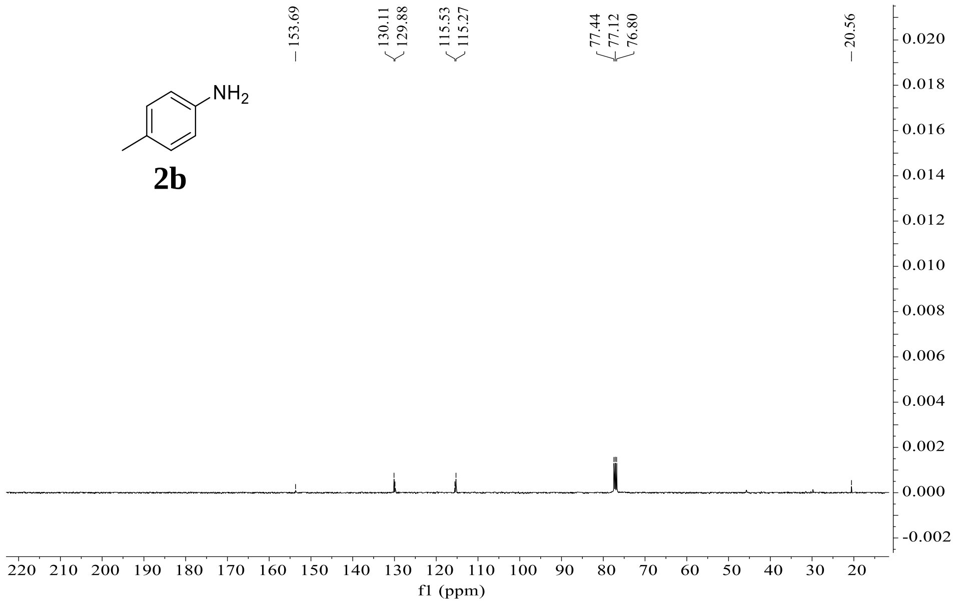

Supplementary Fig. 21 ^{1}H NMR and ^{13}C NMR spectrum of p-toluidine.

Supplementary Note 17 ^{1}H NMR (400 MHz, $CDCl_3$) δ [ppm] 6.97 (d, J = 4.0 Hz, 2H), 6.61 (d, J = 4.0 Hz, 2H), 3.48 (s, 2H), 2.24 (s, 3H); ^{13}C NMR (101 MHz, $CDCl_3$) δ [ppm] 153.69, 130.11, 129.88, 115.53, 115.27, 20.56; GC–MS (EI) *m/z* 106.98, the theoretical value for C_7H_9N is 107.15.

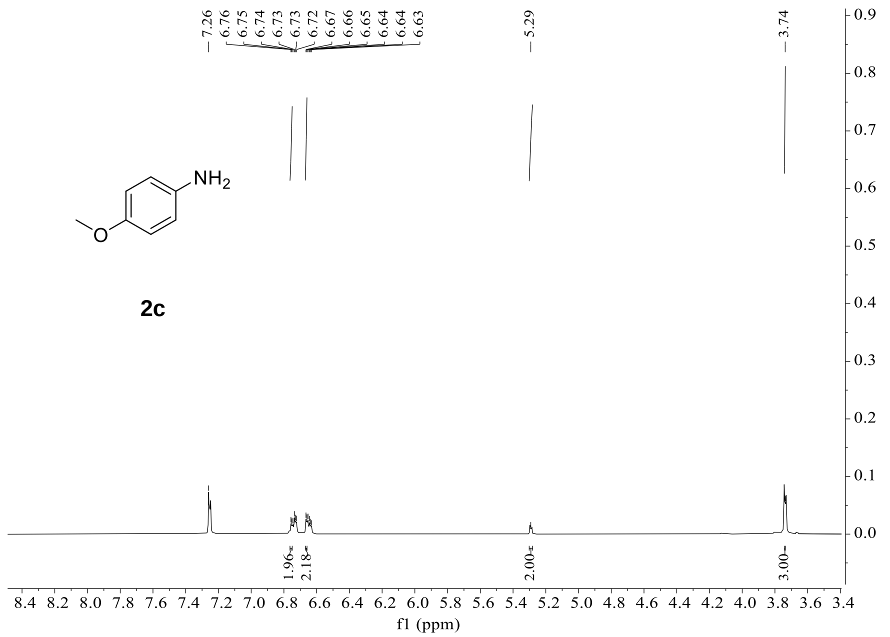

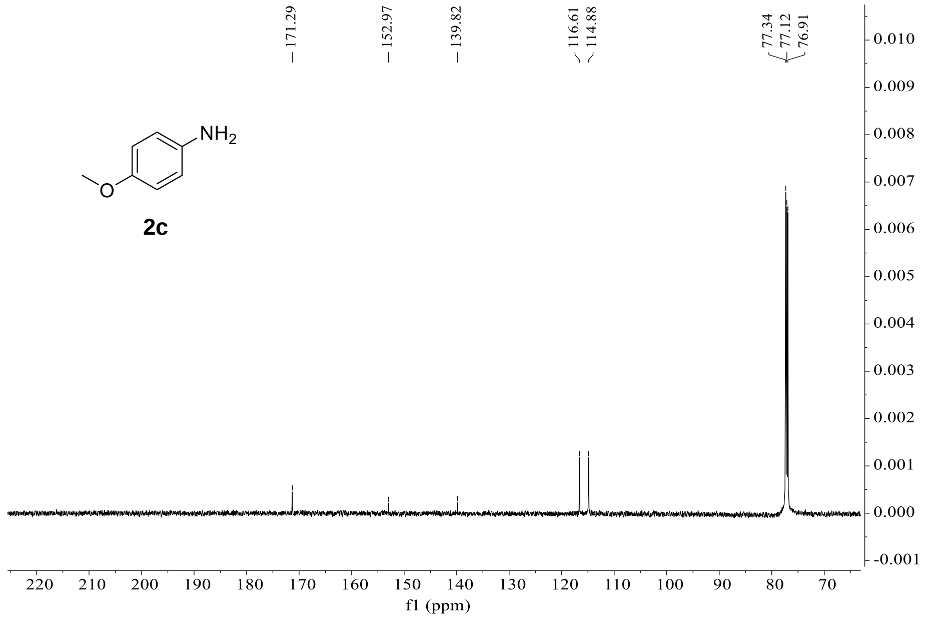

Supplementary Fig. 22 ^{1}H NMR and ^{13}C NMR spectrum of p-anisidine.

Supplementary Note 18 ^{1}H NMR (400 MHz, $CDCl_3$) δ [ppm] 6.76 – 6.72 (m, 2H), 6.67 – 6.63 (m, 2H), 5.29 (s, 2H), 3.74 (s, 3H); ^{13}C NMR (101 MHz, $CDCl_3$) δ [ppm] 171.29, 152.97, 139.82, 116.61, 114.88; GC–MS (EI) *m/z* 123.22, the theoretical value for C_7H_9NO is 123.16.

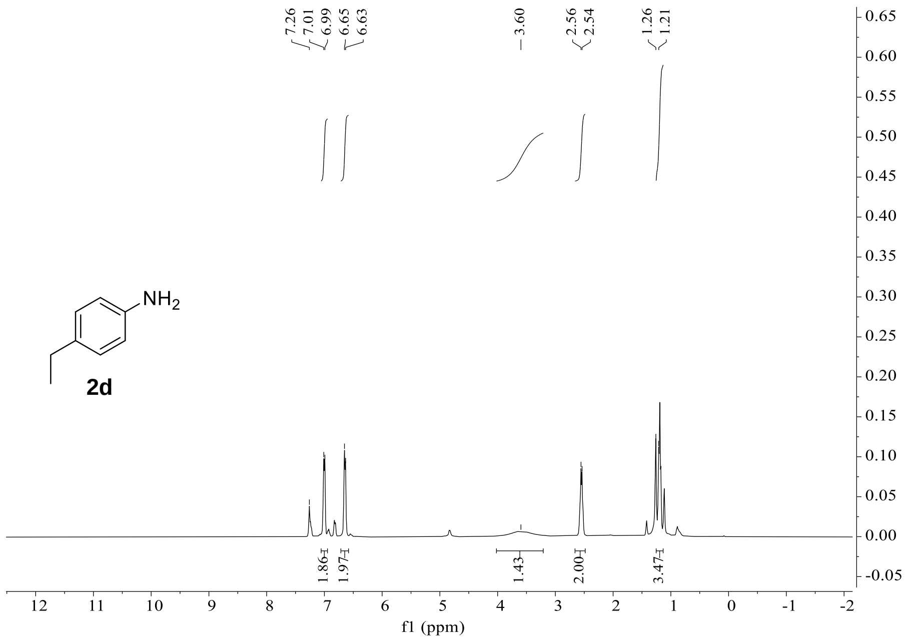

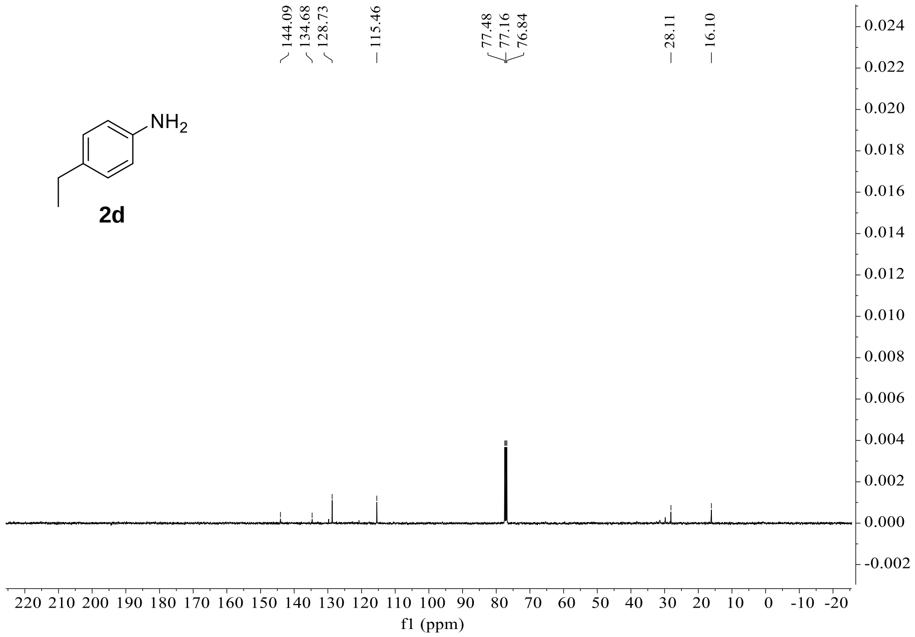

Supplementary Fig. 23 ^{1}H NMR and ^{13}C NMR spectrum of 4-ethylaniline.

Supplementary Note 19 ^{1}H NMR (400 MHz, $CDCl_3$) δ [ppm] 7.00 (d, J = 8.0 Hz, 2H), 6.64 (d, J = 8.0 Hz, 2H), 3.60 (s, 2H), 2.55 (d, J = 8.0 Hz, 2H), 1.26 – 1.21 (m, 3H); ^{13}C NMR (101 MHz, $CDCl_3$) δ [ppm] 144.09, 134.68, 128.73, 115.46, 28.11, 16.10.

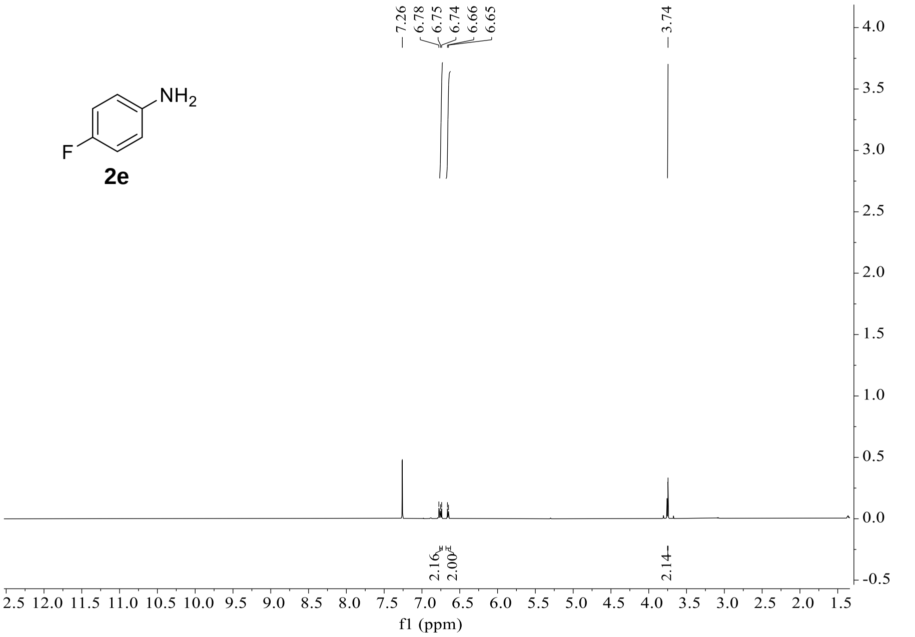

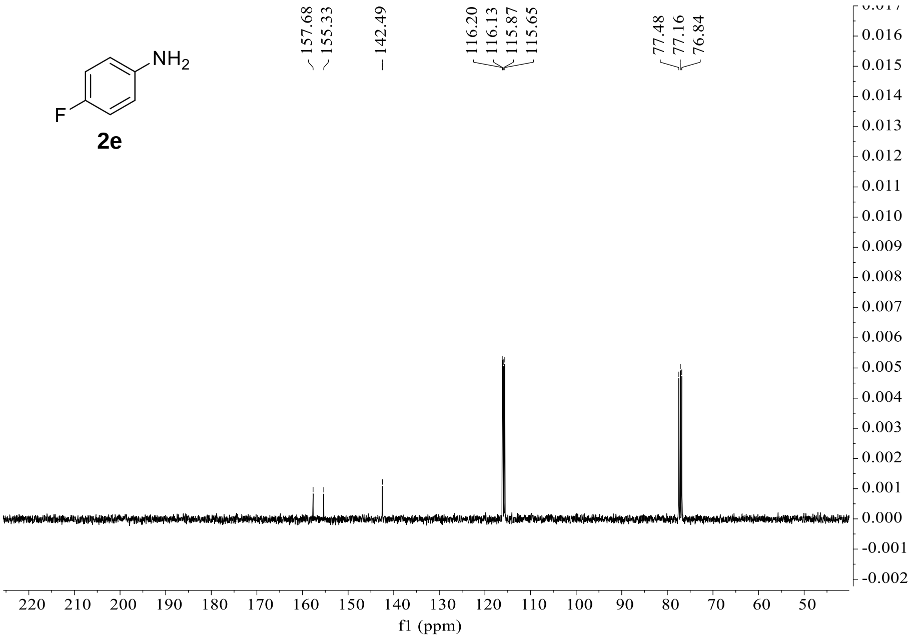

Supplementary Fig. 24 ^{1}H NMR and ^{13}C NMR spectrum of 4-fluoroaniline.

Supplementary Note 20 ^{1}H NMR (400 MHz, $CDCl_3$) δ [ppm] 6.78 – 6.74 (m, 2H), 6.65 (d, J = 4.0 Hz, 2H), 3.74 (s, 2H); ^{13}C NMR (101 MHz, $CDCl_3$) δ [ppm] 157.68, 155.33, 142.29, 116.20, 116.13, 115.87, 115.65.

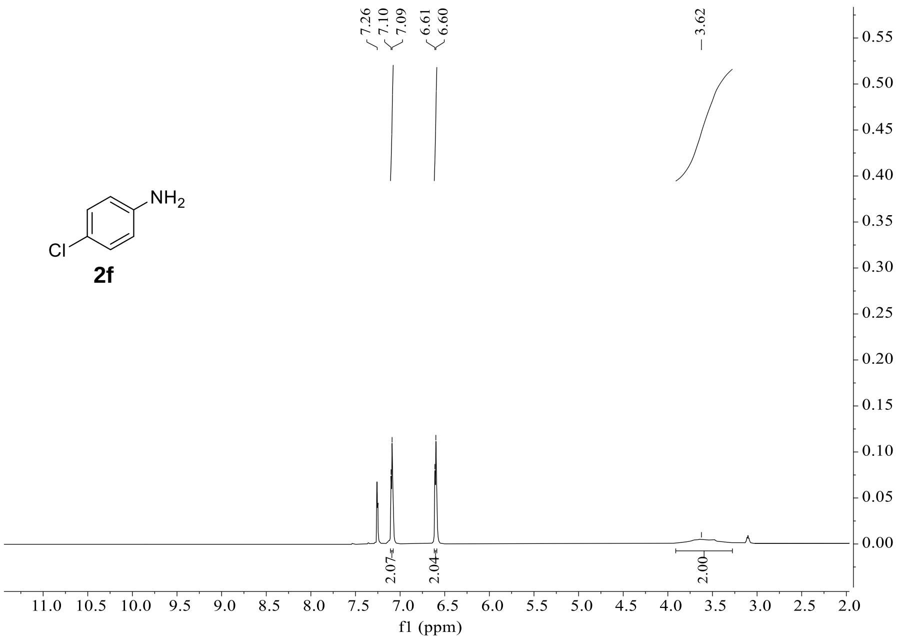

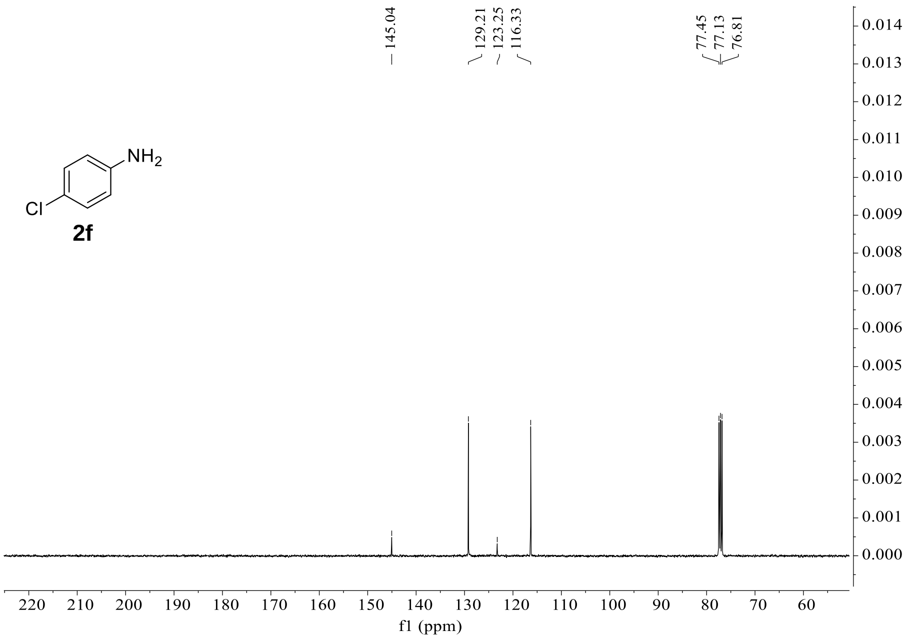

Supplementary Fig. 25 ^{1}H NMR and ^{13}C NMR spectrum of 4-chloroaniline.

Supplementary Note 21 ^{1}H NMR (400 MHz, $CDCl_3$) δ [ppm] 7.09 (d, J = 4.0 Hz, 2H), 6.60 (d, J = 4.0 Hz, 2H), 3.62 (s, 2H); ^{13}C NMR (101 MHz, $CDCl_3$) δ [ppm] 145.04, 129.11, 123.25, 116.33.

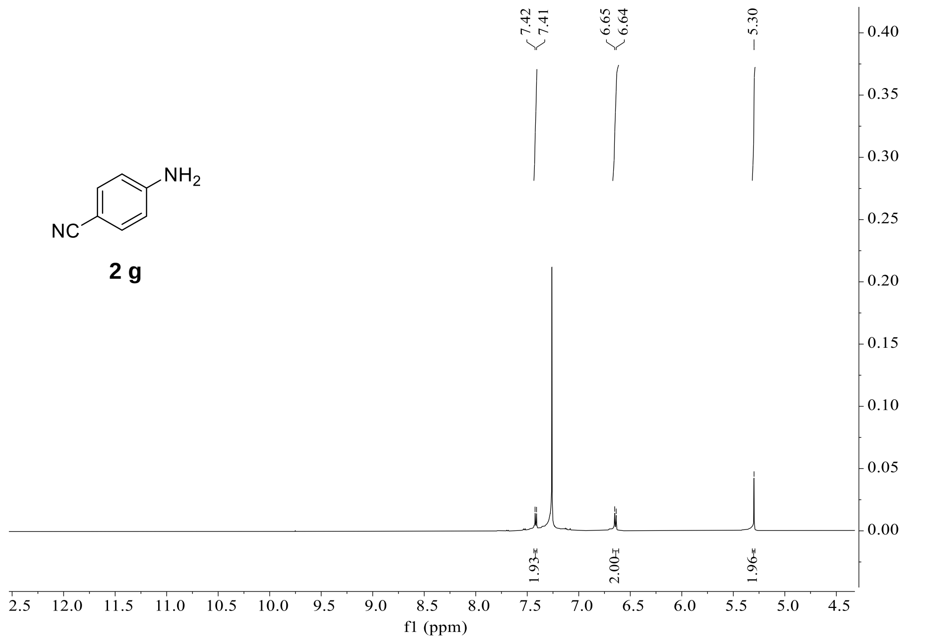

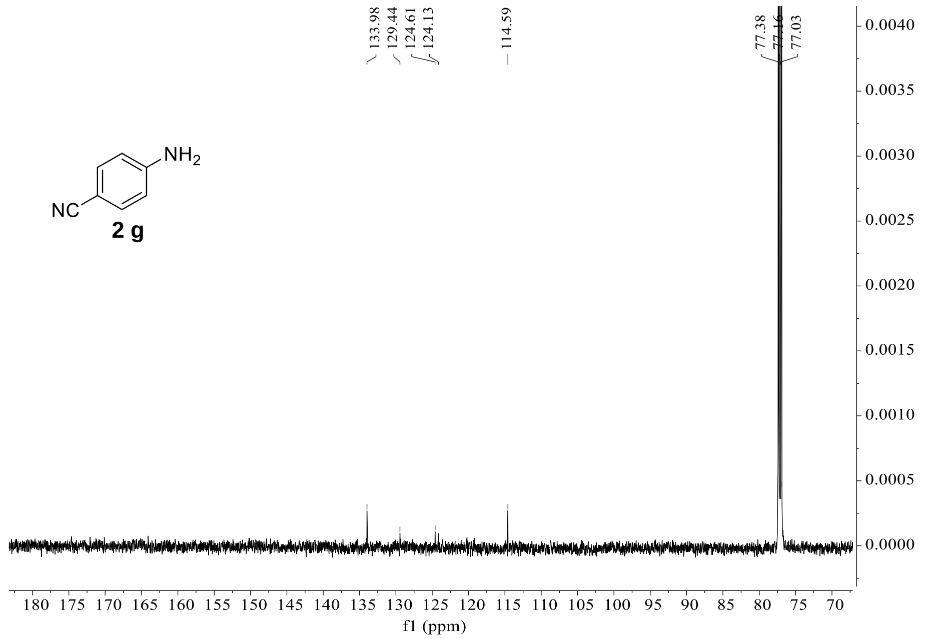

Supplementary Fig. 26 ^{1}H NMR and ^{13}C NMR spectrum of 4-aminobenzonitrile.

Supplementary Note 22 ^{1}H NMR (400 MHz, $CDCl_3$) δ [ppm] 7.41 (d, J = 4.0 Hz, 2H), 6.64 (d, J = 4.0 Hz, 2H), 5.30 (s, 2H); ^{13}C NMR (101 MHz, $CDCl_3$) δ [ppm] 133.98, 129.44, 124.61, 124.13, 116.33.

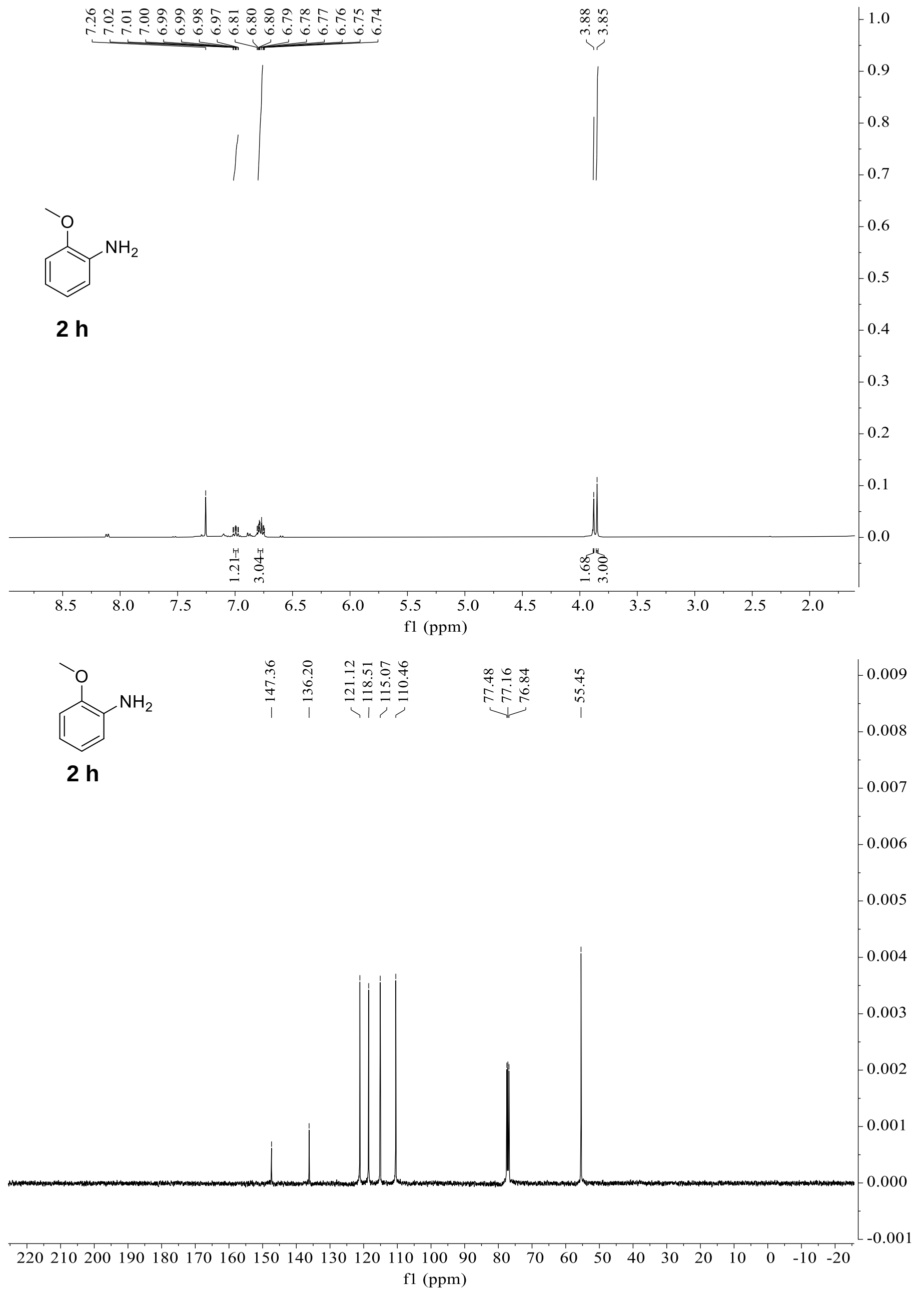

Supplementary Fig. 27 ^{1}H NMR and ^{13}C NMR spectrum of o-anisidine.

Supplementary Note 23 ^{1}H NMR (400 MHz, $CDCl_3$) δ [ppm] 7.02 – 6.97 (m, 1H), 6.81 – 6.74 (m, 3H), 3.88 (s, 2H), 3.85 (s, 3H); ^{13}C NMR (101 MHz, $CDCl_3$) δ [ppm] 147.36, 136.20, 121.12, 118.51, 115.07, 110.46, 55.45.

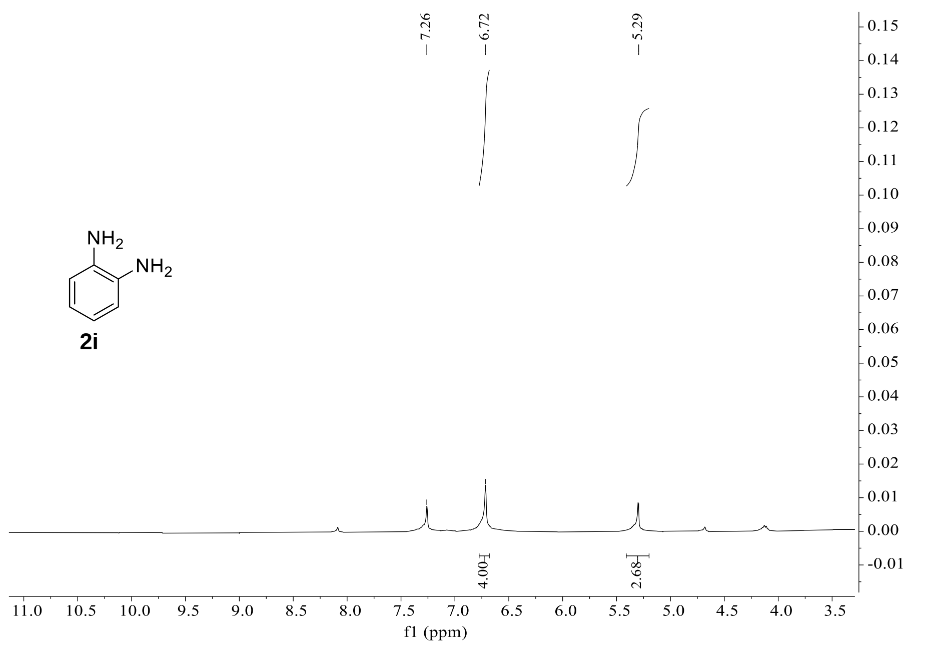

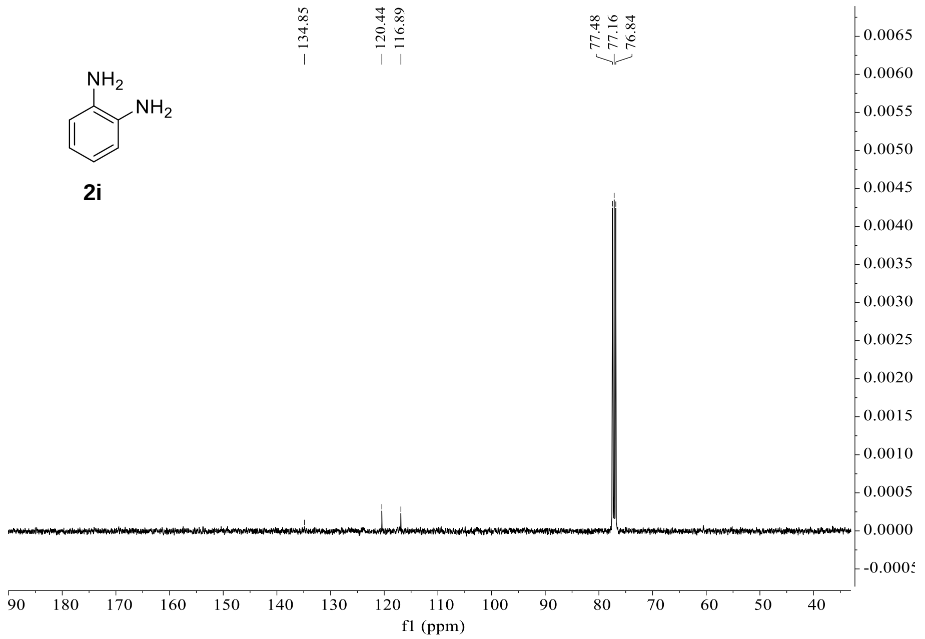

Supplementary Fig. 28 ^{1}H NMR and ^{13}C NMR spectrum of o-phenylenediamine.

Supplementary Note 24 ^{1}H NMR (400 MHz, $CDCl_3$) δ [ppm] 6.72 (s, 4H), 5.29 (s, 4H); ^{13}C NMR (101 MHz, $CDCl_3$) δ [ppm] 134.85, 120.44, 116.89.

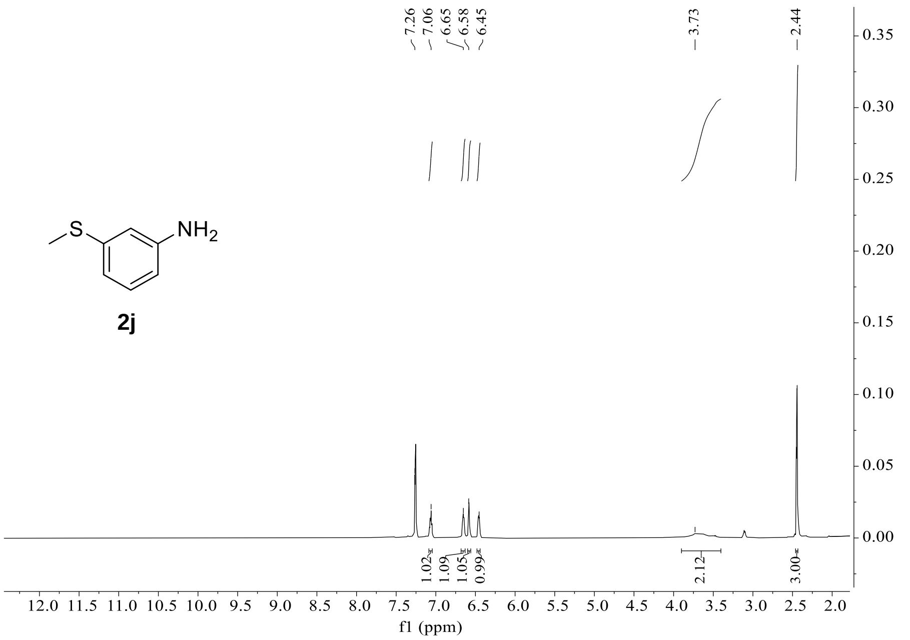

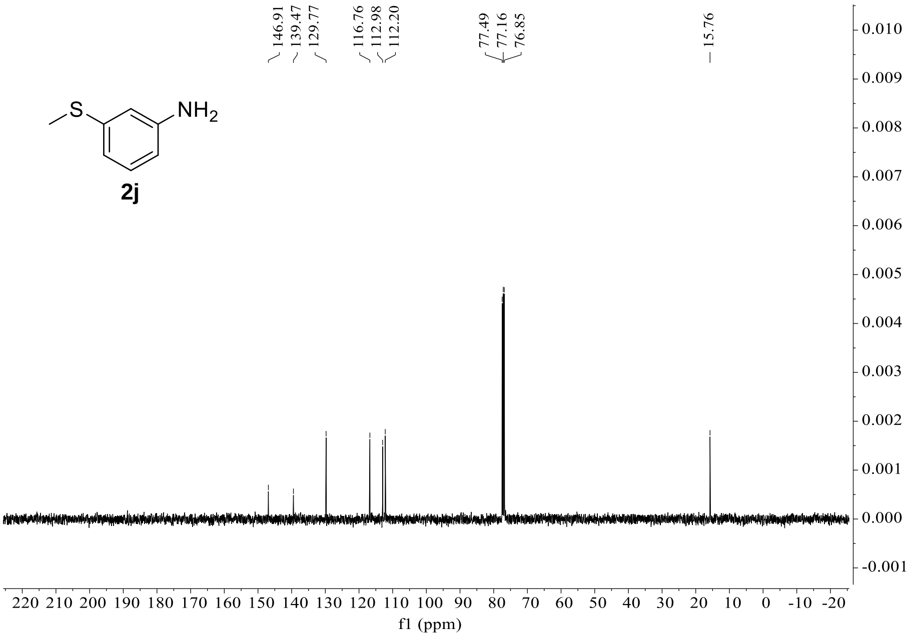

Supplementary Fig. 29 ^{1}H NMR and ^{13}C NMR spectrum of m-methylthioaniline.

Supplementary Note 25 ^{1}H NMR (400 MHz, $CDCl_3$) δ [ppm] 7.06 (s, 1H), 6.65 (s, 1H), 6.58 (s, 1H), 6.45 (s, 1H); ^{13}C NMR (101 MHz, $CDCl_3$) δ [ppm] 146.91, 139.47, 129.77, 116.76, 112.98, 112.20, 15.76.

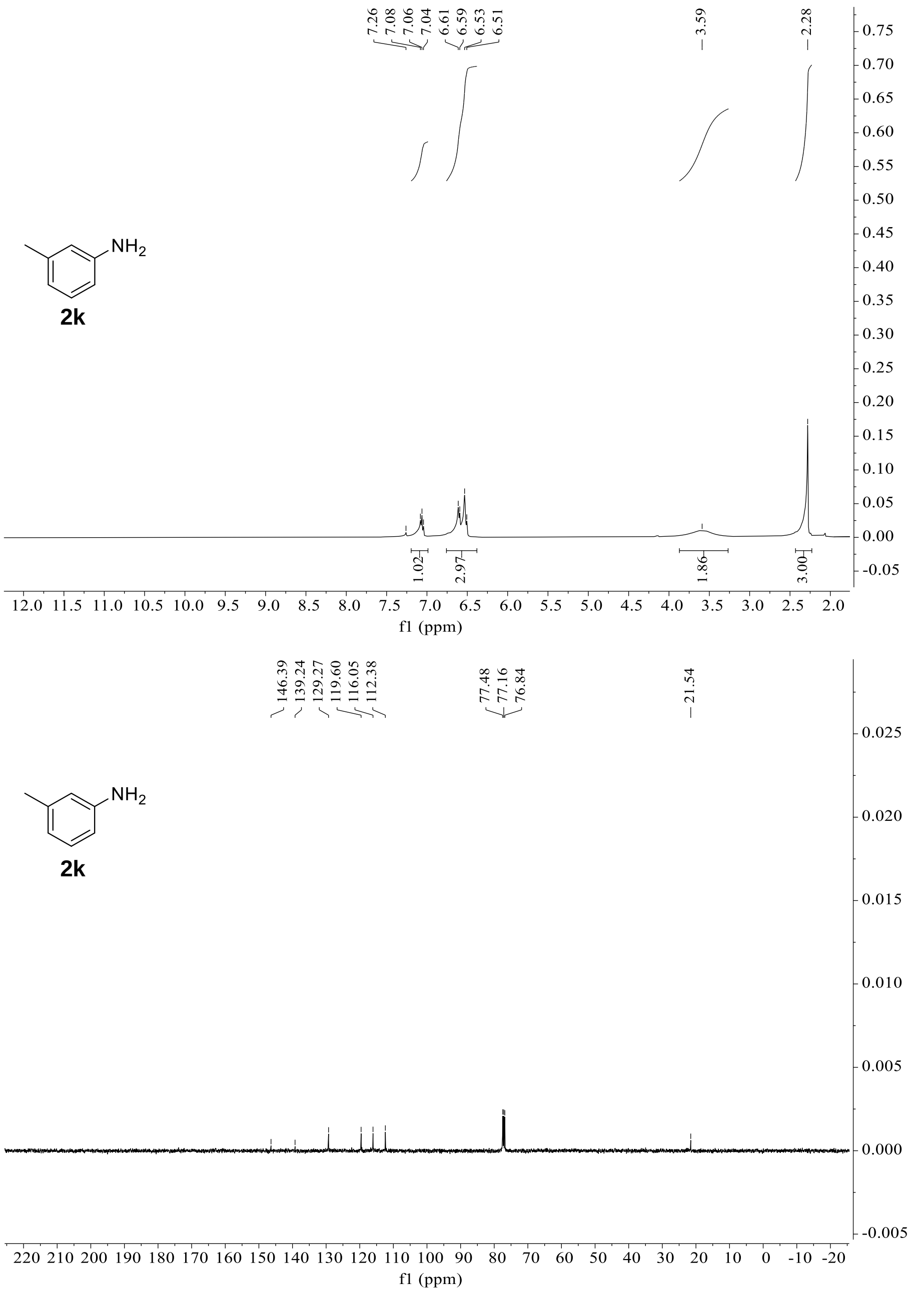

Supplementary Fig. 30 ^{1}H NMR and ^{13}C NMR spectrum of m-toluidine.

Supplementary Note 26 ^{1}H NMR (400 MHz, $CDCl_3$) δ [ppm] 7.08 – 7.04 (m, 1H), 6.61 – 6.51 (m, 3H), 3.59 (s, 2H), 2.28 (s, 3H); ^{13}C NMR (101 MHz, $CDCl_3$) δ [ppm] 146.39, 139.24, 129.27, 119.60, 116.05, 112.38, 21.54.

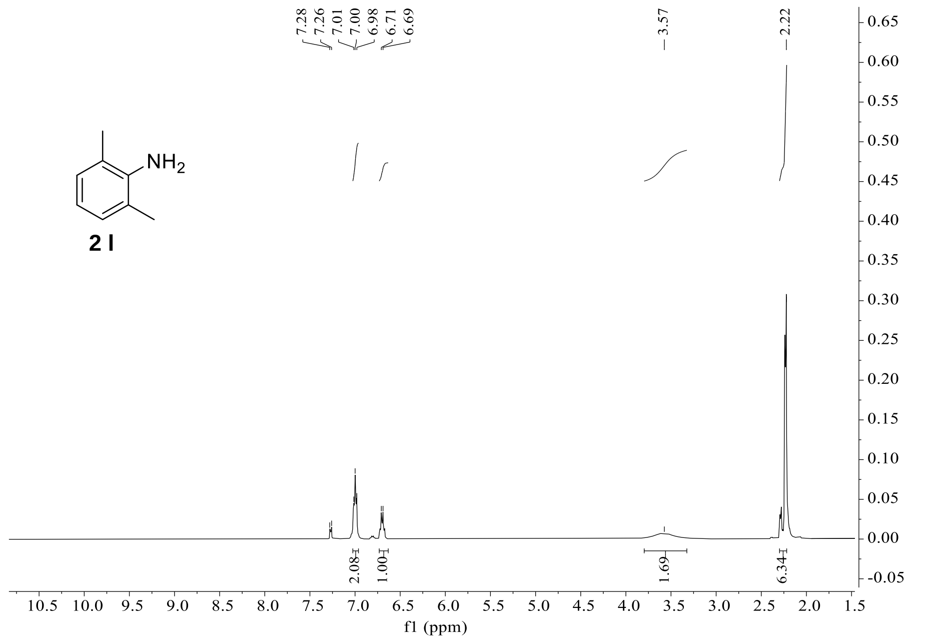

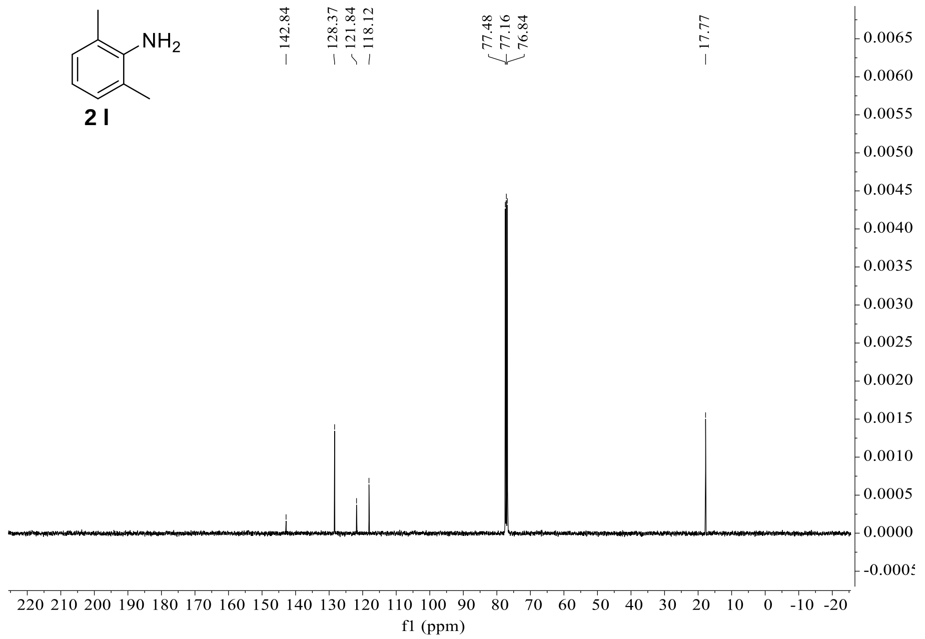

Supplementary Fig. 31 ^{1}H NMR and ^{13}C NMR spectrum of 2,5-dimethylaniline.

Supplementary Note 27 ^{1}H NMR (400 MHz, $CDCl_3$) δ [ppm] 7.01 – 6.98 (m, 2H), 6.70 (d, J = 4.0 Hz, 1H), 3.57 (s, 2H), 2.22 (s, 6H); ^{13}C NMR (101 MHz, $CDCl_3$) δ [ppm] 142.84, 128.37, 121.84, 118.12, 17.77.

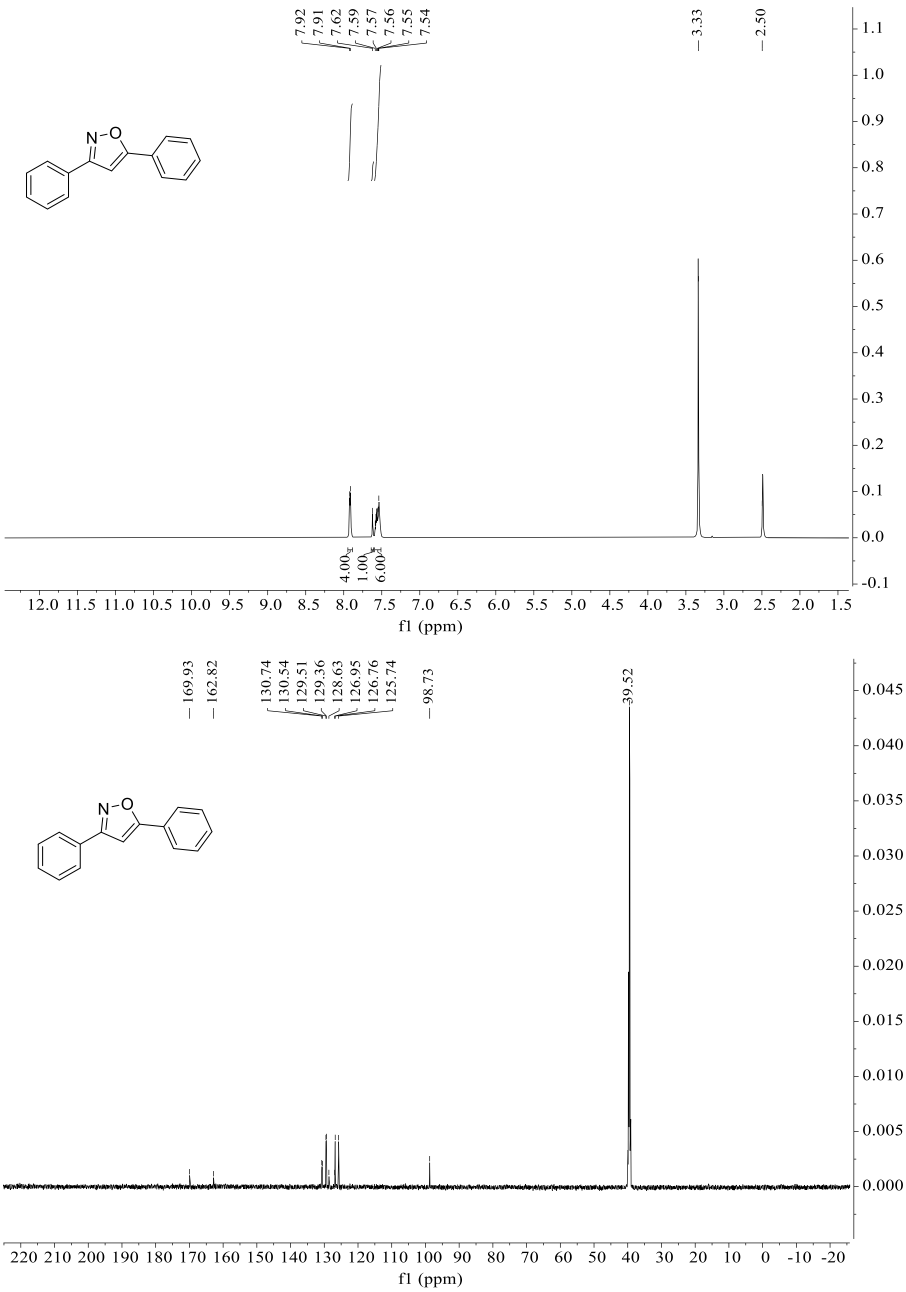

Supplementary Fig. 32 ^{1}H NMR and ^{13}C NMR spectrum of 3,5-diphenylisoxazole.

Supplementary Note 28 ^{1}H NMR (400 MHz, DMSO-*d*6) δ [ppm] 7.92 – 7.91 (d, J = 4.0 Hz, 4H), 7.62 (s, H), 7.59 – 7.54 (m, 6H); ^{13}C NMR (101 MHz, DMSO-*d*6) δ [ppm] 169.93, 162.82, 130.74 (2C), 130.54 (2C), 129.51, 129.36, 128.63, 126.95 (2C), 126.76, 125.74 (2C), 98.73.

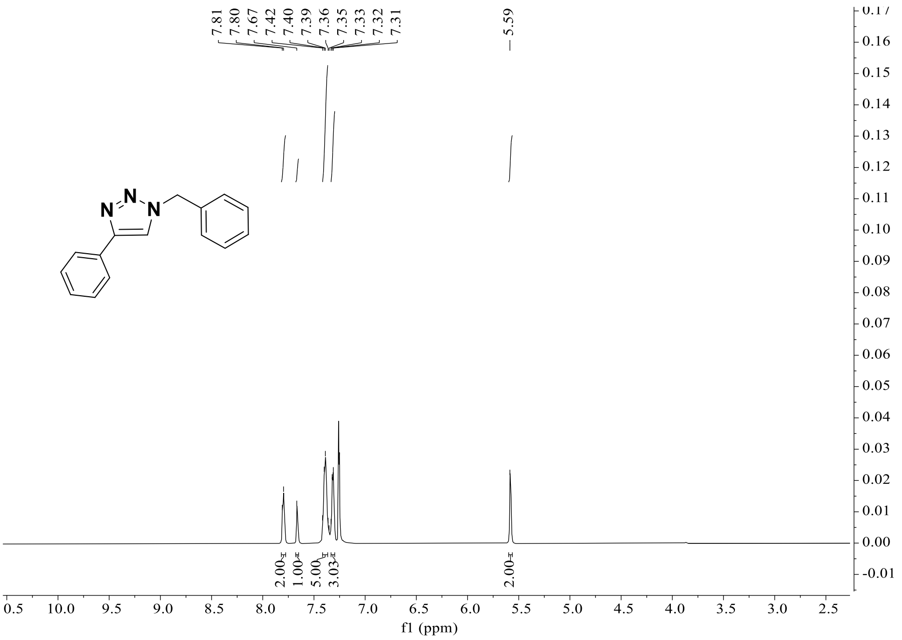

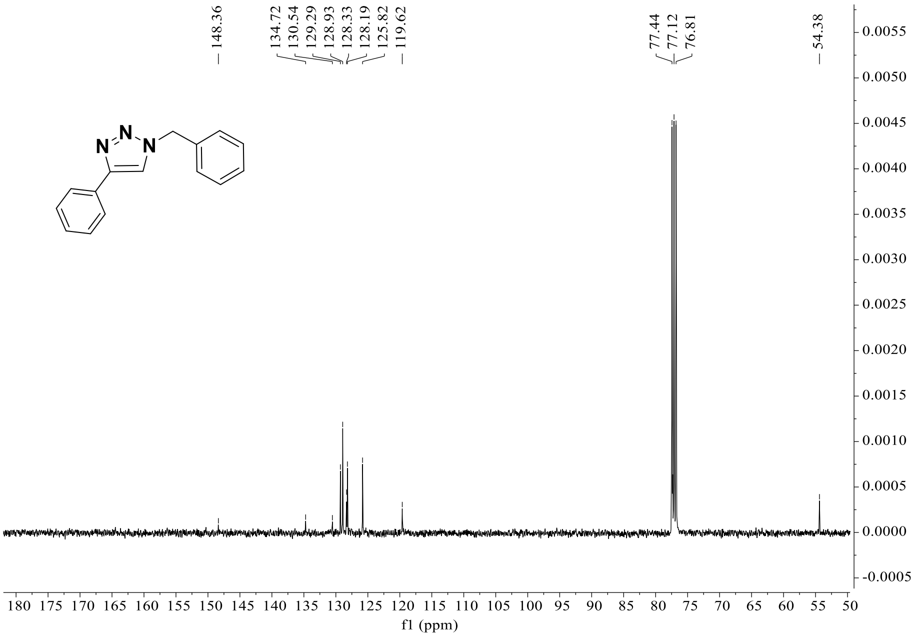

Supplementary Fig. 33 ^{1}H NMR and ^{13}C NMR spectrum of 1-benzyl-4-phenyl-1,2,3-triazole.

Supplementary Note 29 ^{1}H NMR (400 MHz, $CDCl_3$) δ [ppm] 7.80 (d, J = 4.1 Hz, 2H), 7.67 (s, 1H), 7.42 – 7.35 (m, 5H), 7.33 – 7.30 (m, 3H), 5.59 (s, 2H); ^{13}C NMR (101 MHz, $CDCl_3$) δ [ppm] 148.36, 134.72, 130.54, 129.29, 128.93, 128.33, 128.19, 125.82, 119.62, 54.38.

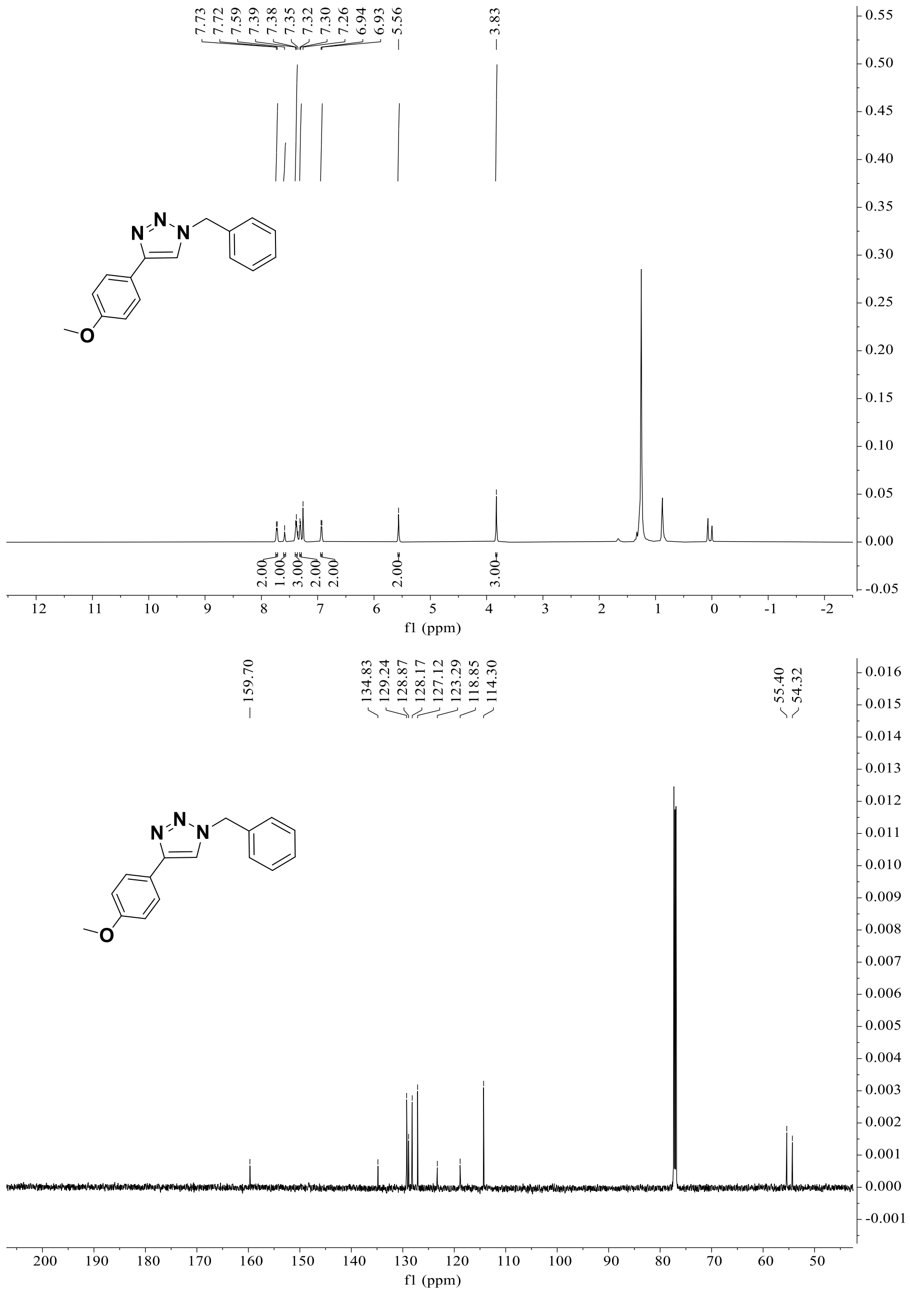

Supplementary Fig. 34 ^{1}**H NMR and** ^{13}**C NMR spectrum of 1-benzyl-4-(4-methoxyphenyl)-1H-1,2,3-triazole.**

Supplementary Note 30 ^{1}H NMR (400 MHz, $CDCl_3$) δ [ppm] 7.73 (d, J = 4.0 Hz, 2H), 7.59 (s, 1H), 7.39 – 7.35 (m, 3H), 7.31 (d, J = 8.0 Hz, 2H), 6.93 (d, J = 4.0 Hz, 2H), 5.56 (s, 2H), 3.83 (s, 3H); ^{13}C NMR (101 MHz, $CDCl_3$) δ [ppm] 159.70, 134.83, 129.24, 128.87, 128.17, 127.12, 123.29, 118.85, 114.30, 55.40, 54.32.

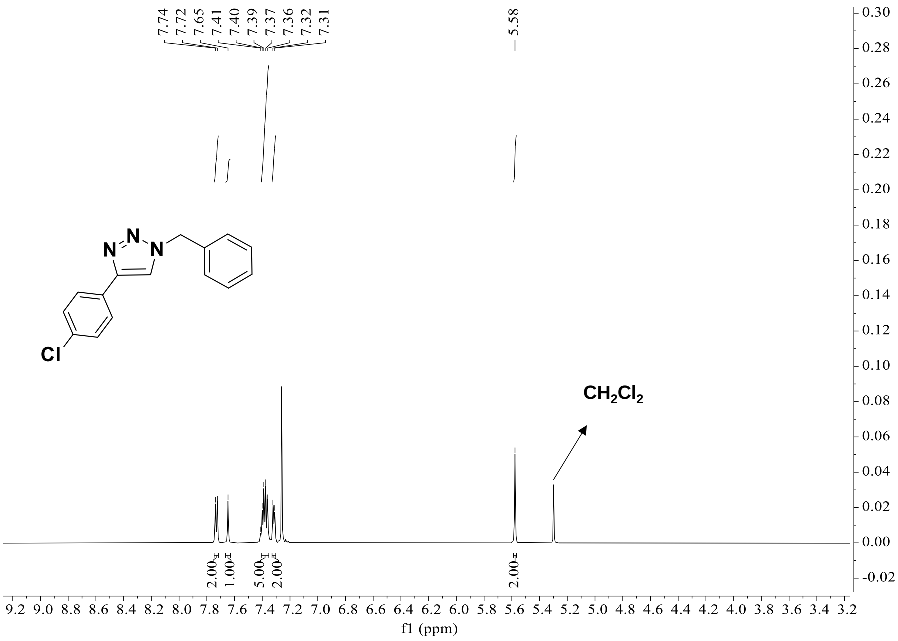

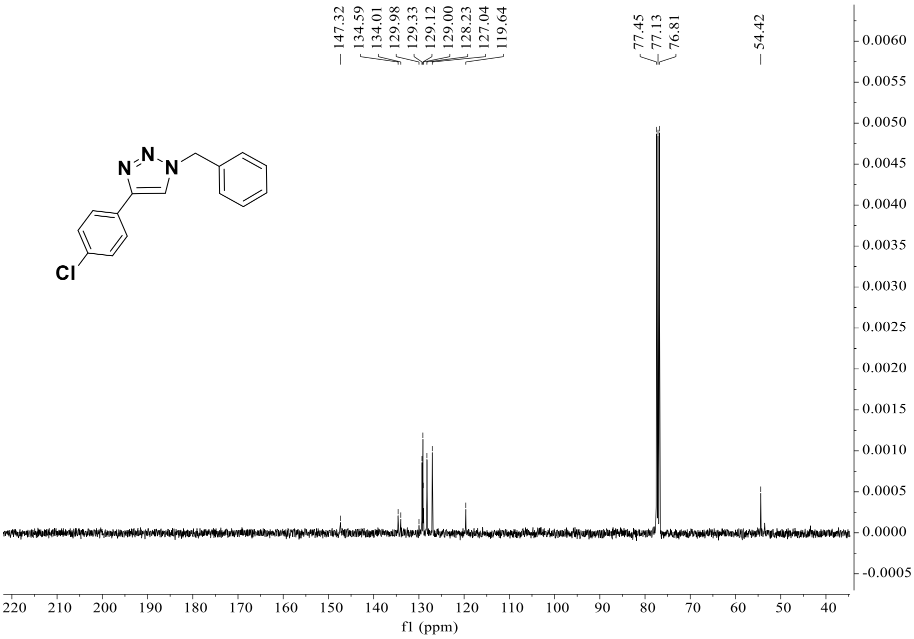

Supplementary Fig. 35 ^{1}**H NMR and** ^{13}**C NMR spectrum of 1-benzyl-4-(4-chlorophenyl)-1H-1,2,3-triazo.**

Supplementary Note 31 ^{1}H NMR (400 MHz, $CDCl_3$) δ [ppm] 7.73 (d, J = 8.0 Hz, 2H), 7.65 (s, 1H), 7.41 – 7.35 (m, 5H), 7.32 (d, J = 4.0 Hz, 2H), 5.58 (s, 2H); ^{13}C NMR (101 MHz, $CDCl_3$) δ [ppm] 147.32, 134.59, 134.01, 129.98, 129.33, 129.12, 129.00, 128.23, 127.04, 119.64, 54.42.

Supplementary References

1. Lam, P., Bonne, D., Vincent, G., Clark, C. & Combs, A. N-Arylation of α-aminoesters with *p*-tolylboronic acid promoted by copper(II) acetate. *Tetrahedron Lett*. **44,** 1691–1694 (2003).
2. Wang, Y., Zhou, W., Jia, R., Yu, Y. & Zhang, B. Unveiling the activity origin of a copper-based electrocatalyst for selective nitrate reduction to ammonia. *Angew. Chem., Int. Ed.* **59,** 5350–5354 (2020).
3. Qiao, W. et al. Paired electrochemical N–N coupling employing a surface-hydroxylated Ni_3Fe-MOF-OH bifunctional electrocatalyst with enhanced adsorption of nitroarenes and anilines. *ACS Catal.* **11,** 13510–13518 (2021).
